# Numerical Simulation of Membrane Separation Characteristics of Supercritical Carbon Dioxide and Water

**DOI:** 10.3390/membranes13120892

**Published:** 2023-11-29

**Authors:** Zongliang Qiao, Yue Pan, Youfei Tang, Yue Cao, Fengqi Si

**Affiliations:** Key Laboratory of Energy Thermal Conversion and Control of Ministry of Education, Southeast University, Nanjing 210096, China; 220210626@seu.edu.cn (Y.P.); 230198055@seu.edu.cn (Y.T.); ycao@seu.edu.cn (Y.C.); fqsi@seu.edu.cn (F.S.)

**Keywords:** supercritical carbon dioxide, membrane–absorption separator, hollow fiber membrane, numerical simulation, separation efficiency

## Abstract

To solve the problem of water carryover in the supercritical CO_2_ separation and mining process in the CO_2_ plume geothermal system, a three-dimensional shell-tube hollow fiber membrane absorption separator is designed in this study. A coupled species transport model, a porous medium model, and an absorption mathematical model are established, and the flow field and separation characteristics in the circular and flat tubes are analyzed using numerical simulation. The results show that the membrane separation efficiency increases with an increase in the flatness and membrane tube length. When the inlet velocity of the mixture is 0.1 m/s, the separation efficiency can reach 75.92%. Selecting a smaller flow Reynolds number and a more significant membrane tube flatness will reduce the water mass fraction at the outlet. When adding baffles of different shapes to the membrane tube, the mixture fluid in the membrane tube meanders forward and flows in the shape of “Z” under the blocking effect of the arcuate baffles. With an increase in the number of arcuate baffles in the membrane tube, the membrane separation efficiency of the separator increases continuously. The mixture fluid flows in the membrane tube with the built-in torsional baffles in a spiral manner, and the separation efficiency of the membrane separator increases with a torsion ratio reduction in the membrane tube.

## 1. Introduction

**Figure 1:**
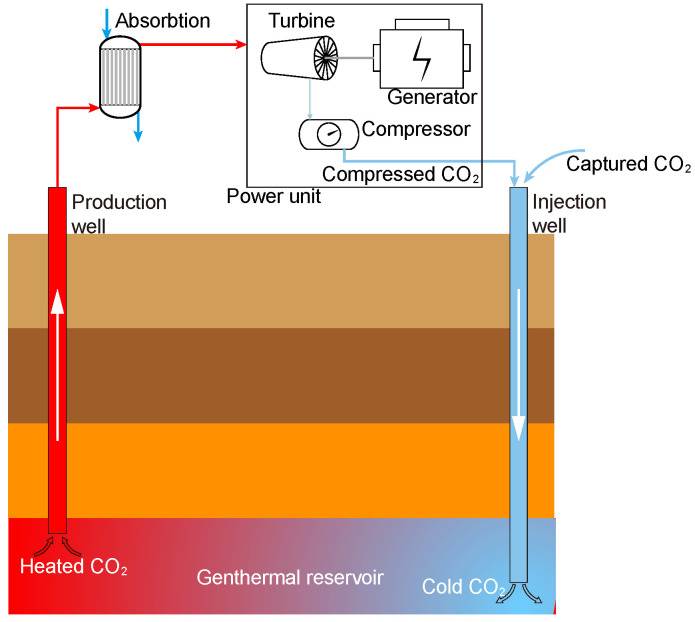
Schematic of CPG system.

Global warming is one of the most critical environmental issues. Fossil fuels account for 81% of global energy consumption, and energy-related CO_2_ emissions account for more than two-thirds of total greenhouse gas emissions [1]. There are several effective ways to reduce greenhouse gas emissions [2]: (1) change the energy structure and increase the clean energy proportion of total energy, (2) improve the energy efficiency utilization to reduce CO_2_ emissions, and (3) capture, comprehensive utilization and geological storage of CO_2_ emissions. A CO_2_ plume geothermal (CPG) system is illustrated in Figure 1. The captured CO_2_ is first compressed and injected into the geothermal reservoir from an artificial well, and then the CO_2_ experiences full diffusion and heat exchange. After the CO_2_ is sufficiently heated and reaches a supercritical state, it is extracted in the production wells to be used as a power generation medium. However, supercritical carbon dioxide (sCO_2_) extracted from the ground will carry much water, which may cause corrosion of the heat exchanger in the CPG power generation system [3], which seriously affects the system’s reliability and service life. Therefore, it is precious to study the separation of the sCO_2_-H_2_O mixture.

Authors have researched separation mechanisms for the scCO_2_-H_2_O mixture in published studies [4,5]. The principles of gravitational and centrifugal force are used to design the axial guide vane cyclone separator and the biconical hydrocyclone. The separation mechanism of the immiscible scCO_2_-H_2_O mixture through numerical simulation is explored. The operating characteristics of these separators are analyzed. The results show that the two separators can achieve a good separation effect. The biconical hydrocyclone can realize the separation of water droplets with a smaller particle size than the axial flow guide vane separator. However, some scholars found that [6,7], when the temperature is 313.15~478.15 K, and the pressure is 10~50 MPa, water will dissolve in sCO_2_, and the mixture cannot be separated. For this separation requirement, other separation methods need to be studied. As sCO_2_ is a solvent with both liquid and gas properties, it diffuses similarly to gases and dissolves similarly to liquids. Low temperature, absorption, and adsorption [8,9,10] may be potential separation technologies for separating water from mixtures. However, the low-temperature technology is expensive, which is inconsistent with the purpose of CO_2_ utilization. The adsorption technology is only effective at low pressure (2~3 MPa). The absorption separation technology will also lead to secondary pollution of the mixture and absorption solution, leading to a more complex separation process. The membrane separation technology is also challenging because of the high-pressure difference on both sides of the membrane during the separation process [11].

**Figure 2:**
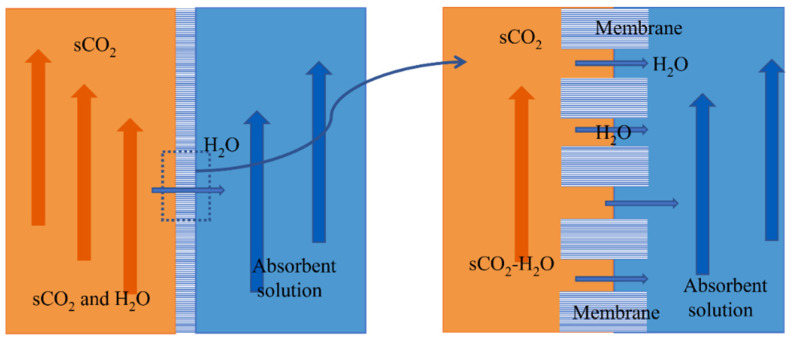
Schematic diagram of sCO_2_-H_2_O miscible fluid membrane absorption and separation process.

Membrane–absorption separation technology combines membrane separation with physical absorption, which is considered a better choice. The membrane absorption process is shown in Figure 2 [12]. It uses a microporous membrane as the mass transfer platform, takes the membrane as a mass transfer site without selectivity, and uses physical absorption to solve the problem of excessive pressure difference on both sides of the membrane. Membrane absorption forms a phase interface through the membrane pore port. A concentration difference drives the water in the membrane pore. It reaches the surface of the absorption solution and then is separated. SCO_2_ is more difficult than vapor to dissolve in a lithium bromide (LiBr) solution. At the same time, LiBr solution has strong moisture absorption and can effectively function at a temperature range of 0~190 °C [13]. Therefore, LiBr solution will be used as the absorbent in this study. Membrane absorption can solve the problem of absorbing solutions carried by supercritical CO_2_. In addition, compared with other separation methods, the process energy loss of membrane absorption separation of scCO_2_ and water-miscible fluid is small, which is conducive to efficiently utilizing supercritical CO_2_ heat carrier fluid.

In the research of membrane separator design, a hollow fiber membrane (HFM) separator can maximize the membrane surface area that a specific volume accommodates, reducing the pressure vessel cost required by the membrane, so it is widely used [14,15,16,17,18,19]. Ouyang and Zhang [16] studied the flow and transfer phenomena in an HFM used for air-dehumidifying liquid desiccants. They used the finite volume method to solve the differential equations of continuity, momentum, energy, and concentration to obtain the shell side mass transfer coefficient, which was verified in experiments. Yoshimune and Haraya [17] studied the permeability of CO_2_-CH_4_ mixed gas through a carbon HFM module using a sulfonated poly (cyclopentyl) (SPPO) derived carbon module. They found that the carbon HFM module can achieve good gas separation performance. Moulin et al. [18] studied the mass transfer in the spiral and straight HFM modules and made a comparison. The results showed that at the same Reynolds number and the mass transfer coefficient, the spiral HFM module was nearly four times higher than that of the straight shape. Wu et al. [19] used numerical simulation to establish various arrangement modes of HFM tubes in the filter channel and analyzed the effects of different arrangement modes and operating parameters, including membrane tube diameter, transverse pitch, and longitudinal pitch of membrane tubes, on the distribution of flow field velocity and pressure.

Researchers have conducted extensive studies on membrane absorption and separation processes using experiments and numerical simulation methods. Quek et al. [20] set up an experimental platform for membrane absorption and separation, used a gas chromatograph to detect the separation effect and studied the CO_2_ separation process from CH_4_ and N_2_. Kang et al. [21] studied the CO_2_ mass transfer of membrane absorption and separation in natural gas through experiments and obtained the rules of the influence of operating variables. Li and Tsotsis [22] built a membrane absorption separation device for the methanol synthesis process and carried out experimental research under different pressure, temperature and absorption liquid flow. Mansourizaheh and Ismail [23] summarized the numerical simulation method of membrane absorption separation for air dehumidification. They established the liquid phase equation, gas phase equation and membrane phase equation by characteristics of the membrane absorption separation to describe the mass transfer process. Lim et al. [24] used numerical simulation to analyze the optimization of the design parameters for the HFM modules. Ma et al. [25] conducted computational fluid dynamics (CFD) simulation modeling the porous medium to simulate the processing process in the HFM, proving the influence of fiber distance and position on the fiber interaction. Wotzka et al. [26] found that according to the mass ratio of H_2_O-CO_2_, the separation of the mixture through the MFI zeolite membrane will be affected. At a low H_2_O-CO_2_ ratio, when the separation factor is lower than 10, the separation performance is poor. The higher the H_2_O-CO_2_ mass ratio, the higher the separation level, which can reach more than 1000.

To sum up, many experiments and simulation studies on membrane absorption and separation are currently carried out, but the object is mixed gas or atmospheric wet air. To the best of the authors’ knowledge, there have been no studies on the separation of sCO_2_-H_2_O mixtures at high temperatures and pressures so far, except for some studies conducted by the authors’ team [12]. It is valuable to study further membrane separation to improve the economics of the process. A three-dimensional membrane absorption separator model is established using the CFD method to address the problem of water carried by sCO_2_ extracted from the ground. Based on the model verification, the membrane separator performance in separating mixtures is explored. At the same time, the effects of different parameters, such as the inlet parameters, the geometric size, the tube shape, and the structure of built-in baffles on the separation efficiency are analyzed in detail. It also provides a possible choice for building the best membrane absorption separator model.

## 2. Numerical Simulation of Membrane Absorption and Separation Process

### 2.1. Design of Membrane Separator

The membrane tube separator we use is similar to the equipment used for absorption refrigeration, and the refrigeration uses condensation as a way to absorb water. In our manuscript, the reason for separating the water vapor from the gas mixture is to prevent the water vapor from liquefying and causing damage to the high-velocity equipment. The primary component of the gas mixture, supercritical carbon dioxide, serves as the working medium for power generation equipment and requires maintenance of a high-energy state. While condensation can enhance water removal efficiency, it also results in heat loss and decreased temperature of the CO_2_, which is not conducive to operation. The goals in designing the separation equipment are to maintain high energy CO_2_ while removing as much vapor as possible.

**Figure 3:**
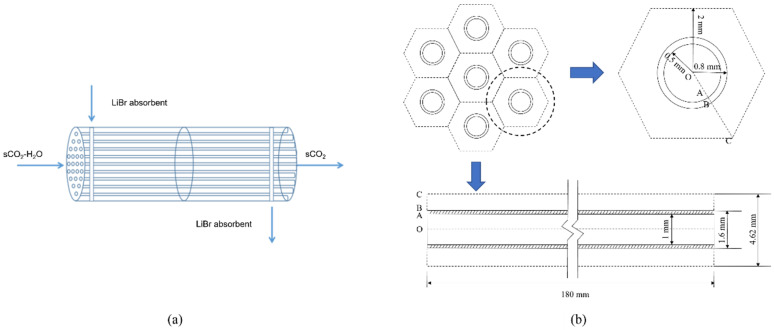
Structure diagram of membrane absorption separator. (**a**) Flow direction diagram of membrane separator; (**b**) Geometric diagram of the HFM module.

The separator structure is similar to a shell-tube heat exchanger, as shown in Figure 3a. Water containing sCO_2_ flows in the tube, and LiBr absorption liquid flows outside the tube and is separated using the countercurrent flow. Considering that the tube’s shape and internal structure will impact the mass transfer process, the membrane module selected in this study is a circular tube and a flat tube. Based on the smooth tube structure, a bow baffle-type spoiler is installed inside, and the impact on the separation performance before and after optimization is compared and analyzed. The numerical simulation method is used to study the membrane absorption and separation process, as well as the change rule of separation efficiency with the parameters of sCO_2_ and LiBr absorption liquid and the structural parameters of the separator. Because the structural types of each channel of the membrane tube are the same, this study only selects a single-channel membrane tube for numerical analysis. The selected simulation object is shown in Figure 3b. The cross-section of the separation unit of each membrane tube is regarded as a regular hexagon [12]. According to the number of membrane tubes arranged in this section, it can calculate the size of the hexagon.

### 2.2. Numerical Model

Based on the ANSYS software platform, the coupled species transport model, porous medium model, and water absorption mathematical model are established by numerical simulation. A three-dimensional model of an HFM separator is established, and the flow field with separation in the membrane domain is simulated. To simplify the modeling, the following assumptions need to be made in the simulation process. (1) The mass transfer is steady. (2) The both-side flow of the sCO_2_-H_2_O mixture and LiBr absorption solution is laminar. (3) The heat generation in the absorption is ignored. (4) LiBr solution and sCO_2_-H_2_O mixture have the same temperature.

#### 2.2.1. Single-Phase Porous Medium

The governing equation for general scalar transport in isotropic porous media in single-phase flow is

(1) $$\frac{\partial }{\partial t}\left(\gamma \rho \phi \right)+\nabla \cdot \left(\gamma \rho \vec{v}\phi \right)=\nabla \cdot \left(\gamma \Gamma \nabla \phi \right)+\gamma S_{\phi },$$

where γ is the porosity, ρ is the density, $\vec{v}$ is the velocity, Γ is the diffusivity, ϕ is the scalar quantity, and $S_{\phi }$ is the ϕ-related source term.

The volume-mean mass and momentum equations are

(2) $$\frac{\partial }{\partial t}\left(\gamma \rho \right)+\nabla \cdot \left(\gamma \rho \vec{v}\right)=0,$$

(3) $$\frac{\partial }{\partial t}\left(\gamma \rho \vec{v}\right)+\nabla \cdot \left(\gamma \rho \vec{v}\vec{v}\right)=-\gamma \nabla p+\nabla \cdot \left(\gamma \vec{\tau }\right)+\gamma \vec{B}-\left(\frac{\mu }{\alpha }+C_{2}\rho \left|\vec{v}\right|\right)\vec{v}.$$

The last term in Equation (3) represents the viscous resistance and inertial resistance.

#### 2.2.2. The Porous Medium Model

The FLUENT component transport model is used to simulate the HFM. It can obtain the influence of porous media using an additional momentum source term to the flow equation. This momentum source term has two terms, viscosity, and inertial loss, and the expression is

(4) $$S_{i}=-\left(\sum_{j=1}^{3}D_{ij}\mu \nu_{j}+\sum_{j=1}^{3}C_{ij}\frac{1}{2}\rho \left|\nu \right|\nu_{j}\right),$$

where i=x,y,z represents the direction, μ is the dynamic viscosity, D is the viscosity resistance coefficient, and C is the inertial loss coefficient.

For homogeneous porous media, the source term is

(5) $$S_{i}=-\left(\frac{\mu }{\alpha }\nu_{i}+C_{2}\frac{1}{2}\rho \left|\nu \right|\nu_{i}\right),$$

where α is the permeability.

Darcy’s law can calculate the laminar pressure drop of fluid in porous media.

(6) $$\nabla p=-\frac{\mu }{\alpha }\vec{\nu },$$

where p is the pressure. The Ergun equation gives the viscosity resistance 1/α as

(7) $$\frac{1}{\alpha }=\frac{150{\left(1-\lambda \right)}^{2}}{D_{P}^{2}\lambda^{2}}.$$

where $D_{P}^{}$ is a mean grain diameter.

#### 2.2.3. Mathematical Model of Water Absorption

Water forms a phase interface between the absorption solution and the gas mixture for the absorption model. The water absorption capacity of the LiBr solution depends on the vapor pressure of water on its surface ($P_{w,\nu }$) and the partial pressure of water in the sCO_2_-H_2_O mixture ($P_{w,m}$). Therefore, the driving force of mass transfer for water absorption $\Delta F_{w}$ can be expressed as

(8) $$\Delta F_{w}=\frac{P_{w,m}}{P_{m,i}-P_{w,m}}-\frac{P_{w,\nu }}{P_{m,i}-P_{w,\nu }}>0,$$

where $P_{w,m}$ can be calculated by Dalton’s partial pressure law in kPa, $P_{w,\nu }$ can be estimated by the saturation temperature of the water $T_{w,sat}$ in kPa, and $T_{w,sat}$ can be calculated using NIST REFPROP 9.0 [27] in °C.

The Dooling gives the relationship as $T_{w,sat}$ and the temperature of the LiBr solution $T_{S}$ as

(9) $$T_{S}=A_{D}T_{w,sat}+B_{D},$$

where $A_{D}$ is the Turin slope, and $B_{D}$ is the Turin intercept. When the concentration of the LiBr absorption solution is 65%, $A_{D}=1.2$, $B_{D}=55$ °C [13]. In the membrane absorption separator, the water in the sCO_2_-H_2_O mixture will be absorbed by the solution, and the separation efficiency (η) is calculated as

(10) $$\eta =\frac{{\dot{m}}_{s,i}-{\dot{m}}_{s,o}}{{\dot{m}}_{s,i}}\times 100\%,$$

where ${\dot{m}}_{s,i}$ is the inlet water flow rate, and ${\dot{m}}_{s,o}$ is the outlet water flow rate.

### 2.3. Grid Division and Boundary Conditions

#### 2.3.1. Simulation of Membrane Tube Structure Dimensions

The flat tube selected in this study is rolled from a typical smooth circular tube as the base tube; its two ends are semicircular arcs, and the middle section is a parallel plate. Four types of flat tube membrane separator models with different specifications are selected, and the circular tube and flat tube membrane separator are simulated at the same time. The membrane separator length is 180 mm, the radial section radius is 10 mm, and the number of facial mask tubes is 5. Table 1 gives the structural parameters of membrane tubes of different specifications, mainly including the length, width, height, flatness, hydraulic diameter, membrane thickness, and flow area.

#### 2.3.2. Grid Division of Membrane Separator

**Figure 4:**
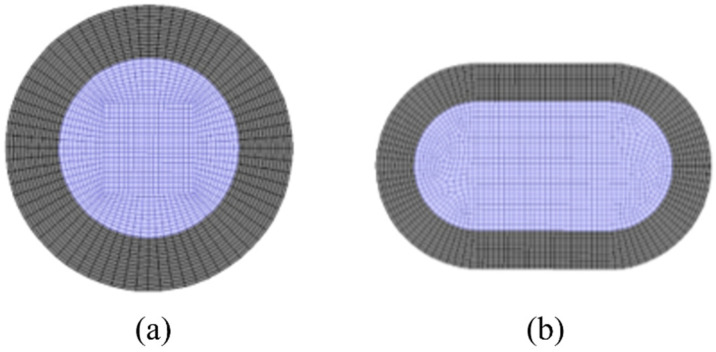
Schematic diagram of grid division of membrane tube radial section. (**a**) Round tube; (**b**) Flat tube. Grey: membrane; purple: fluid mixture.

According to the structural parameters of the membrane tubes mentioned above, the three-dimensional physical model of the membrane tubes of the separator is first established using the ANSYS software platform, and each physical model is grid divided. The grid is divided into unstructured grids from surface to volume. Considering the particularity of fluid flow near the wall, the wall grid is densified. The cross-section is divided into quadrilateral grids. Figure 4 shows the grid division of the radial section of the membrane tube.

**Figure 5:**
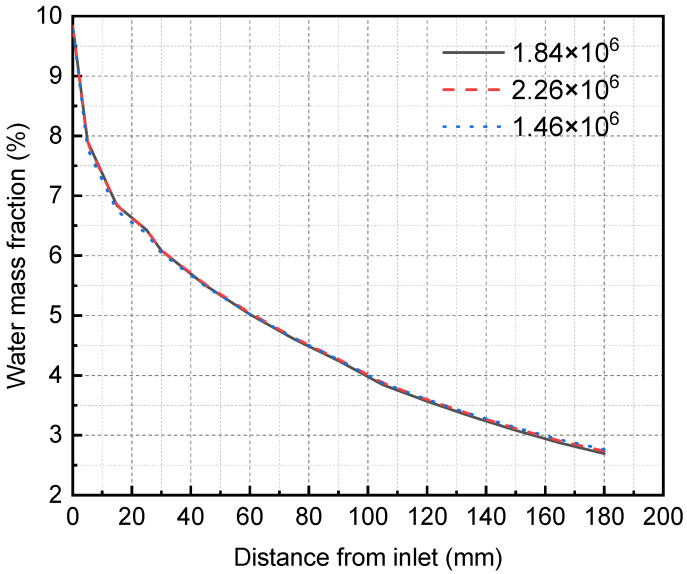
Grid independence verification results of membrane separator.

Because the calculation accuracy may vary with the grid number, the grid independence test must be carried out first to determine the appropriate grid number. The Ansys meshing module divides the structured grid of the three-dimensional membrane separator model and verifies the grid independence using three different unit numbers. The simulation results are shown in Figure 5. In the case of the grid numbers 1.46 × 10^6^, 1.84 × 10^6^, and 2.26 × 10^6^, the grid size does not affect the simulation results, so the total number of grids selected in this study is 1.84 × 10^6^. Specifically, the membrane, mixture flow, and solution flow areas are 1.8 × 10^5^, 5.22 × 10^5^, and 1.14 × 10^6^.

#### 2.3.3. Boundary Conditions

The inlet of the sCO_2_-H_2_O mixture and the LiBr solution is set as the velocity boundary. The outlet of the sCO_2_-H_2_O mixture and the LiBr solution is set as the pressure outlet, the thick layer of the HFM tube is set as the porous medium layer, calculated by the laminar flow model, and other parameters adopt the system default values. The specific boundary condition parameters are shown in Table 2, in which the LiBr solution concentration of 65% by a mass fraction is used because of the high temperature at the inlet of the membrane separator. The ice crystal formation is inhibited [28], and the mass fraction of the sCO_2_-H_2_O mixture is determined by the solubility of H_2_O in sCO_2_ [29].

### 2.4. Model Validation

**Figure 6:**
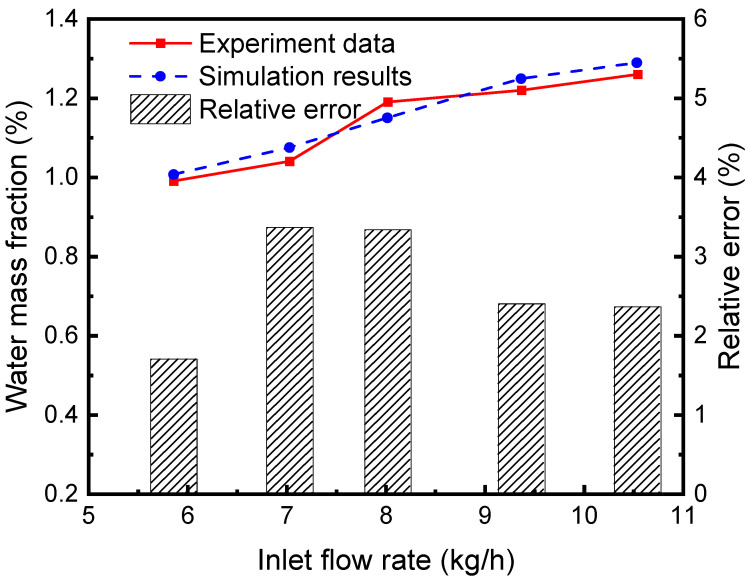
Comparison between simulation results and experimental data [30].

The separation efficiency of the continuous air dehumidification system is simulated and analyzed. A representative unit consisting of liquid desiccant flow inside a single hollow fiber and airflow outside the tube is studied. Simulation is carried out according to the established HFM separator model, and the proposed model’s accuracy is verified by comparing it with the experimental results in reference [30]. As can be seen from the comparison of simulation and experimental results and their relative errors in Figure 6, the average relative error of the water fraction at the outlet of the separator (the ratio of water mass in the mixed fluid to the mixed fluid mass) is 2.64%. These simulated data agree well with experimental data, indicating the accuracy of the model in simulating the absorption and separation of the sCO_2_-H_2_O mixture in the HFM tube by the LiBr solution.

## 3. Simulation Results and Analysis

### 3.1. Flow Field Analysis

**Figure 7:**
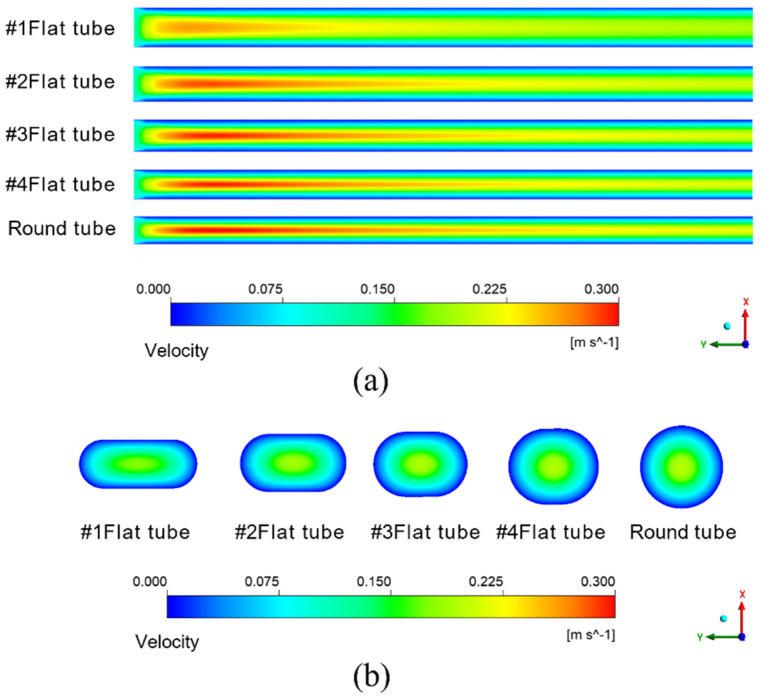
Velocity distribution of different membrane tubes. (**a**) XY section of membrane tubes with different specifications; (**b**) Radial section of membrane tube outlet with different specifications.

The simulation results given in Figure 7a are under the condition that the inlet velocity of the mixture is 0.1 m/s. The temperature is 433.15 K. It means the schematic diagram of mixed fluid flow velocity in membrane tube membrane separators with different specifications on xy plane under the same inlet flow rate, it can be seen that with a continuous increase in the flow distance of the mixed fluid in the membrane tube, the flow velocity of the fluid first reaches the maximum near the inlet of the membrane tube, and the maximum velocity is about 0.3 m/s and then decreases gradually with an increase in the flow distance of the mixture due to the influence of friction resistance. Simultaneously, when the membrane tube’s flatness decreases continuously, the friction resistance in the tube decreases. Along the fluid flow direction, the flow velocity of the mixture fluid in the membrane tube decreases gradually, and the outlet velocity of the fluid increases. The simulation results given in Figure 7b are the velocity distribution diagrams of the radial sections at the outlet of membrane tube separators of different specifications. The center velocity of the membrane tube is the highest. The velocity gradually decreases along the radial direction of the membrane tube. With a decrease in the flatness of the membrane tube, the velocity at the center gradually increases. Because the tube is flatter, the upper and lower walls are closer to the center of the tube, so the velocity gradient between the relative flat walls increases significantly.

**Figure 8:**
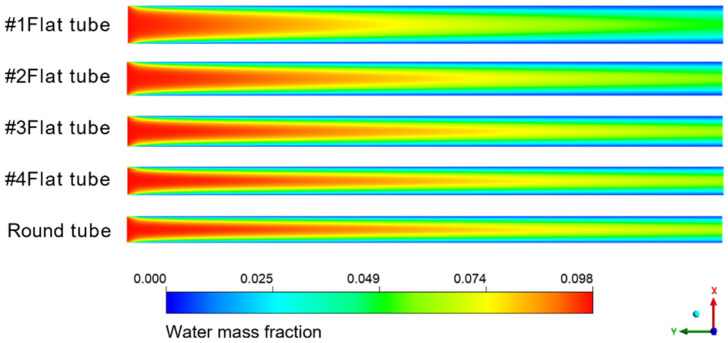
Water mass fraction distribution in XY section of membrane tubes of different specifications.

Figure 8 shows the cloud diagram of the distribution of water mass fraction in different membrane tubes. Along the fluid flow direction, when the flatness of the membrane tube increases, the water mass fraction at the outlet section of the membrane tube decreases because the increase in the membrane tube flatness will change the similar diameter of the membrane tube. The contact area between the mixed flatness and the absorption liquid will increase, making the separation process more complete, thus improving the separation efficiency. From the normal view of the flow direction, the water mass fraction at the side near the membrane surface is always less than the middle. Because the closer the membrane tube surface is, the closer the mixture fluid is to the LiBr absorption liquid, the greater the driving force of water absorption, and the easier the mixture is to be separated. Therefore, the water mass fraction at both sides of the membrane tube surface is lower, and this trend is more obvious with the increase in the flow distance.

### 3.2. The Influence of Reynolds Number on Separation Efficiency

**Figure 9:**
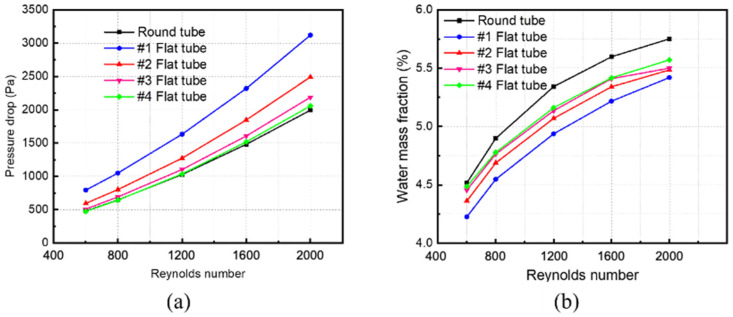
Effect of Reynolds number on parameters of membrane separator. (**a**) Pressure drop of membrane tube; (**b**) mass fraction of water at membrane tube outlet.

Changing the inlet velocity of the mixed fluid and keeping other parameters unchanged simulates and calculates membrane tube separators of different specifications. The Simulation in Figure 9a obtains variations in pressure drop in different-sized membrane tube separators with the Reynolds number. The pressure drop of five kinds of HFM tubes increases with an increase in the Reynolds number, whether at low or high Reynolds numbers. Among them, at the same Reynolds number, the pressure drop in the round tube is the smallest, and that in the #1 flat tube is the largest. The greater the flatness of the membrane tube, the greater the pressure drop of the corresponding membrane tube because the equivalent diameter of the membrane tube changes when the circular tube is replaced by a flat tube. When the same Reynolds number changes, the equivalent flat tube diameter decreases, and the fluid in the corresponding tube is easier to reach the turbulent state, thus increasing the fluid pressure loss in the tube and increasing the corresponding pressure drop.

Figure 9b shows the relationship between the mass fraction of water at the outlet of membrane tube separators of different specifications and the Reynolds number. With a constant increase in the Reynolds number, the outlet water mass fraction of all the HFM tubes increases continuously. When the Reynolds number increases to a higher level (Re > 1600), the increasing trend of the water mass fraction at the outlet of membrane tubes decreases. For the same Reynolds number, the round tube has the highest water mass fraction, while the #1 flat tube has the lowest. The greater the flatness of the flat tube, the smaller the outlet water mass fraction because when the flat tube replaces the round tube at the same Reynolds number, the equivalent diameter of the membrane tube changes. The more excellent the flat tube flatness, the greater the contact area, promoting the contact separation process, thus reducing the water mass fraction at the membrane separator outlet. Although the flat tube increases the contact area at a high Reynolds number, the mixture cannot be fully separated due to the fast fluid flow velocity, so the corresponding separation efficiency increases.

### 3.3. Effect of Mixture Inlet Velocity

**Figure 10:**
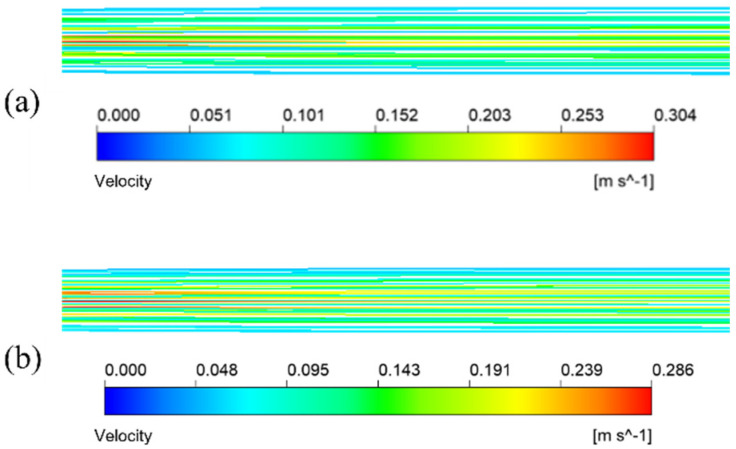
Fluid velocity streamline of the mixture in membrane tube. (**a**) Round tube; (**b**) #2 flat tube.

Figure 10 illustrates the streamlined diagram of the fluid in the circular tube and flat tube under the same mixture inlet velocity. From the diagram, the flow path of the mixture fluid in the two kinds of tubes, in which the fluid flow path in the flat tube and the ordinary circular shape is parallel to the tube axis, and the fluid flow velocity in the circular tube is faster.

**Figure 11:**
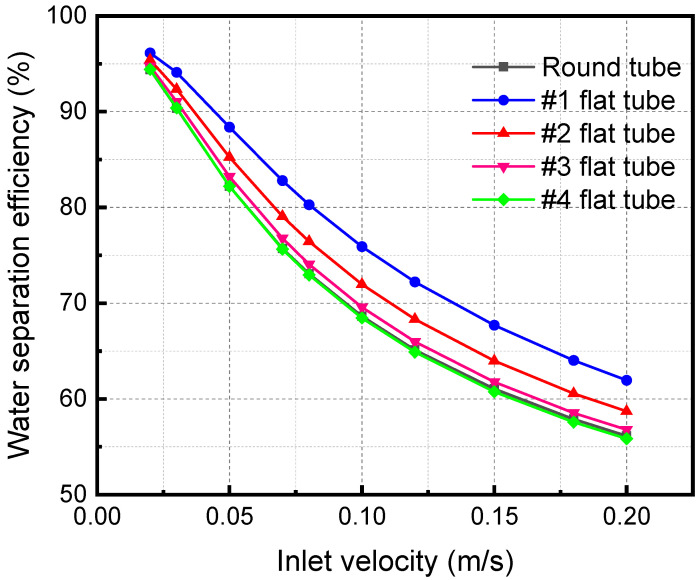
Effect of inlet velocity with different specifications on separation efficiency.

Figure 11 shows the separation efficiency variations with the mixture inlet velocity of the membrane separator. The water flux is an important metric, and the amount that is absorbed away is readily available once the separation efficiency and water vapor flow rate are obtained. Therefore, the change in separation efficiency is the focus of the analysis. The separator separation efficiency decreases with a rise in the mixture inlet velocity. When the mixture inlet velocity is 0.2 m/s, the separation efficiencies of #1, #2, #3, and #4 flat tubes and circular tube membrane separators are 61.95%, 58.70%, 56.78%, 55.85%, and 56.15%, respectively. When the inlet velocity increases, the contact time between the LiBr solution and the gas mixture is shortened. The absorption separation process of the two fluids becomes insufficient, leading to the decline of the membrane separation efficiency. In addition, when the flatness of the membrane tube gradually increases, the separation efficiency gradually increases. When the flatness of the membrane tube increases, the contact area increases under the same mixture inlet flow. It also makes the separation more complete, correspondingly improving the separation efficiency.

### 3.4. The Influence of Membrane Tube Length

**Figure 12:**
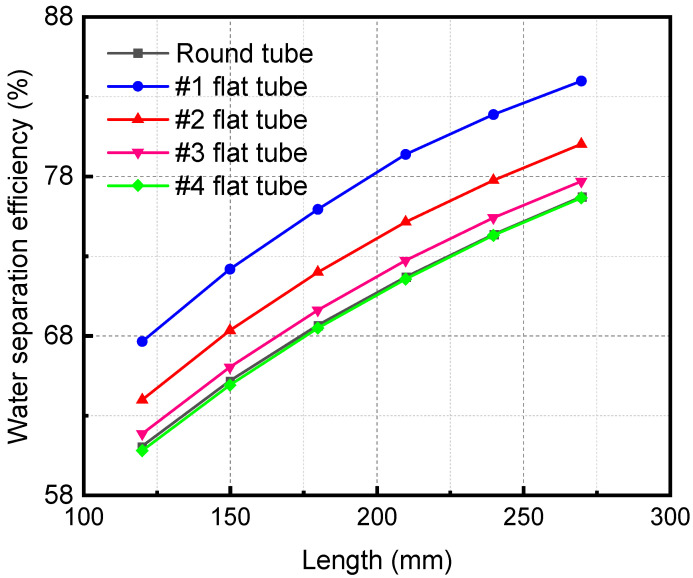
Effect of length of flat tube membrane separators with different specifications on their separation efficiency.

Figure 12 demonstrates the influence of the HFM flat tube length on the separation efficiency under the same mixture inlet flow. The membrane separation efficiency increases with a lengthening of the flat tube. When the membrane tube flatness increases, the separation efficiency also increases. When the length of the membrane separator is 270 mm, the separation efficiency of the #1 flat tube is the highest, 83.98%. As the length of the HFM flat tube increases, the contact area and time are improved. The contact separation of the two fluids is complete, thus raising the separation efficiency. When the tube flatness increases, the contact area expands under the same mixture inlet flow, which also makes the separation more sufficient and the separation efficiency higher.

## 4. Numerical Simulation of Separation Efficiency with Bow Baffle

Adding a spoiler structure in a smooth membrane tube is a significant method to improve the separation efficiency. The purposes of setting the baffle are to increase the turbulence intensity of fluid in the tube, strengthen the separation process between fluids, and improve the separation efficiency of the separator. A spoiler does not need to change the separator’s overall structure. Many baffle structures can be used to optimize the structure of the hollow fiber separator, among which the bow baffle can improve the cross-flow effect, enhance the disturbance, and thus improve the separation efficiency. Meanwhile, the bow baffle is cheap and easy to manufacture. In this study, arch baffles are added to the circular tube to keep the basic structural parameters of the membrane separator unchanged. The length is 180 mm, the radial section radius is 10 mm, and the inner and outer radii of the membrane are 0.5 mm and 0.8 mm, respectively. Arch baffles with half of the flow area are added. The number of arch baffles is variable, and the five membrane separators with baffle spacing of 45 mm, 36 mm, 30 mm, 18 mm, and 12 mm are simulated.

### 4.1. The Influence of Bow Baffle

**Figure 13:**
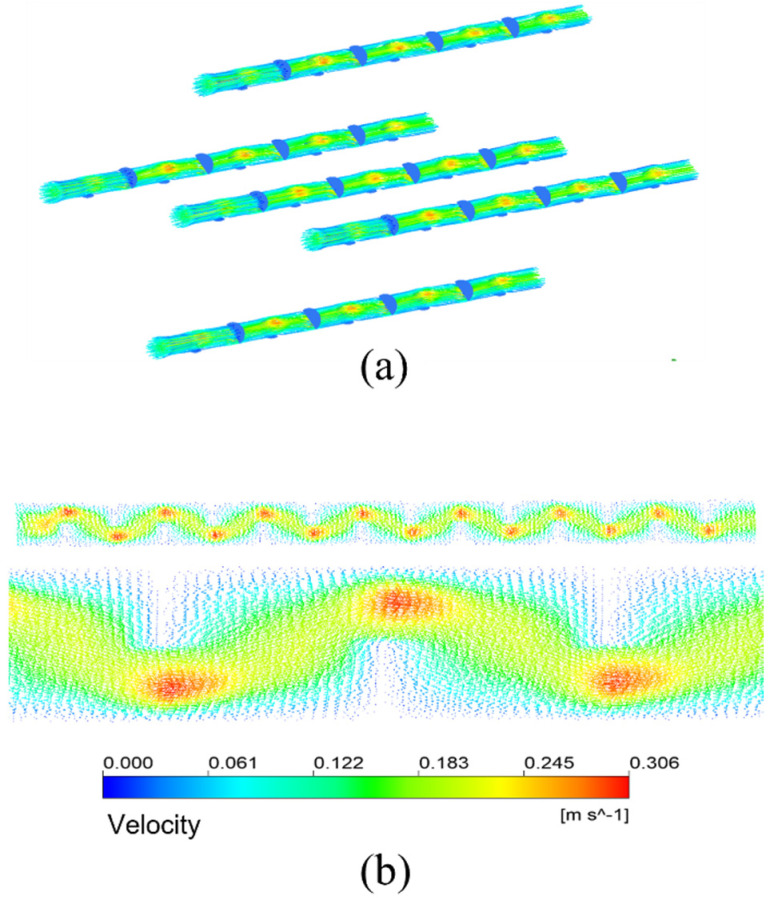
Streamline and velocity distribution vector in membrane tube of membrane separator. (**a**) Velocity streamlines; (**b**) Velocity distribution vector.

Figure 13a shows the streamline diagram of the mixture fluid velocity in the membrane tubes. The fluid mixture flows up and down in the tube, and the fluid velocity increases significantly near the baffle. Figure 13b is the velocity distribution vector diagram on the tube side. After the fluid enters the tube side from the inlet, it passes through the area under the baffle plate and meanders forward under the blocking of the baffle plate, showing a “Z” shaped flow. The enlarged view is the velocity vector distribution in the tubular near the front of each baffle plate. The flow direction is almost perpendicular to the baffle plate. In contrast, the flow area below the baffle plate is the downstream. The fluid flow here is parallel to the flow of the tube bundle. The flow velocity is also higher than that between the baffles. The area behind the baffle plate is a low-velocity region. There may be backflow, generating several small vortices. The fluid here is almost static, forming a detention zone.

**Figure 14:**
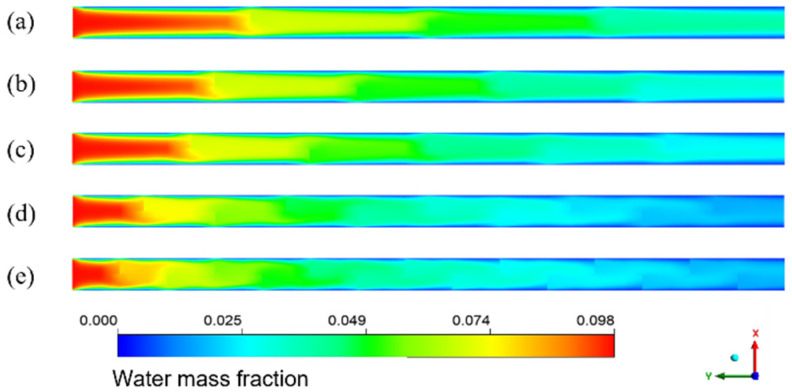
Water mass fraction distribution for different baffle spacings in XY flat facial mask separator. (**a**) 45 mm; (**b**) 36 mm; (**c**) 30 mm; (**d**) 18 mm; (**e**) 12 mm.

The simulation results given in Figure 14 are the water mass fraction distribution of the HFM flat tube separator under the same mixture inlet flow. The number of bow baffles is 3, 4, 5, 9, and 14, respectively. From the inlet to the outlet of the membrane tube, the color of the water mass fraction decreases gradually from dark to light overall. The water mass fraction changes slowly between the flow basins of the two adjacent segmental baffles. In contrast, the mass fraction changes dramatically in the flow area close to the segmental baffles. With a reduction in baffle plate spacing, the decreasing trend of water mass fraction increases—the more baffles, the lower the water mass fraction at the outlet. The number of baffles increases, the flow velocity between baffles increases, and the low-velocity area on the back of each baffle decreases, which means the dead zone decreases. With the reduced baffle plate spacing, the channel area between them becomes smaller, the flow around the fluid becomes more intense, and the fluid in the shell side closer to the vertical tube bundle. The turbulence degree increases, making the fluid contact with the LiBr absorption liquid more sufficiently, and the water mass fraction decreases faster.

**Figure 15:**
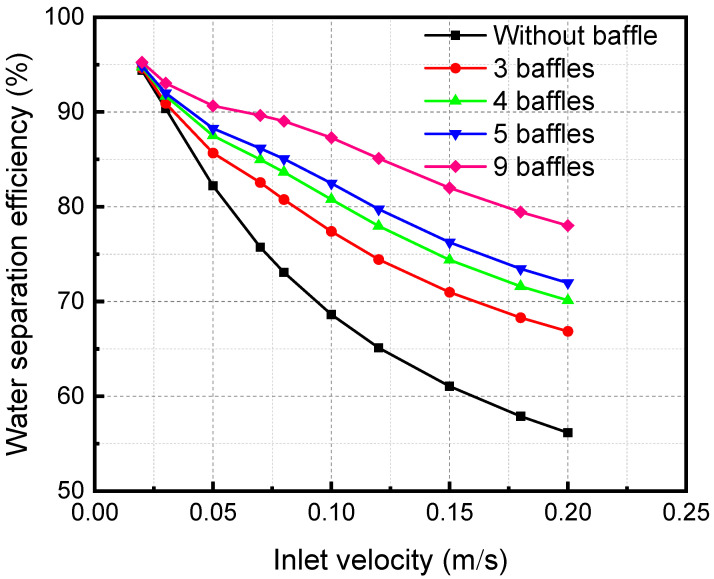
Effect of the inlet velocity of the membrane separator mixture on the separation efficiency under different numbers of baffles.

Figure 15 shows the influence of inlet velocity on separation efficiency when there are different segmental baffles. The separation efficiency decreases with the growth of the inlet velocity. Because an increase in the inlet velocity of the mixture decreases the contact time, the separation becomes inadequate, leading to a reduction in the separation efficiency. As the baffle number increases, the distance between baffles decreases. The separation efficiency increases continuously when the baffle number increases. The disturbance in the fluid flow process increases, which also makes the separation process more adequate and increases the separation efficiency.

### 4.2. Influence of Spiral Bow Baffle

**Figure 16:**
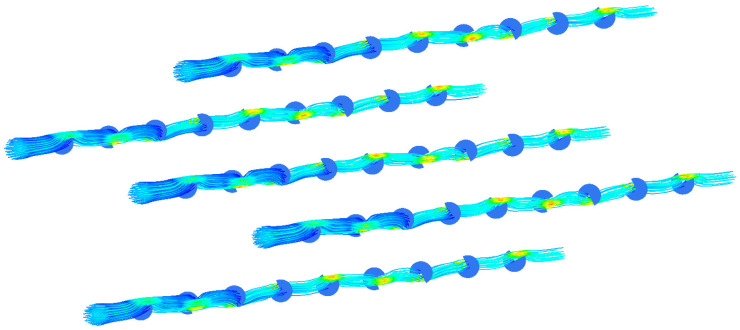
Velocity streamlines in membrane tube.

**Figure 17:**
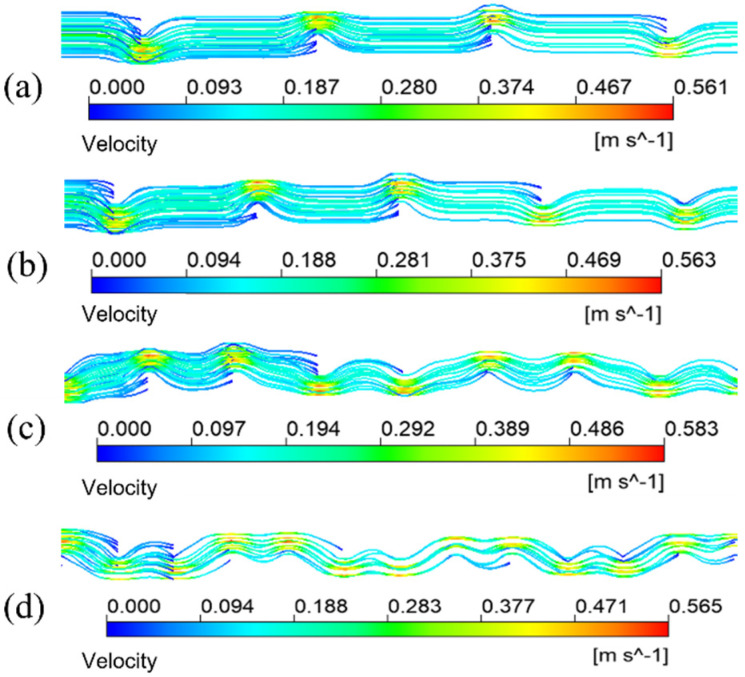
Streamlines of different fluid velocities. (**a**) 4 baffles; (**b**) 5 baffles; (**c**) 9 baffles; (**d**) 14 baffles.

This study gives another arrangement of bow baffle, which uses a spiral arrangement to make the fluid spiral flow in the membrane tube. Figure 16 shows the streamline diagram of the mixture fluid velocity in the membrane tube. It can be seen from the diagram that the mixture of fluid flows in a spiral shape in the tube, and the fluid velocity increases significantly near the baffle. Figure 17 shows the velocity streamline diagram of the mixture fluid when the baffle number is 4, 5, 9 and 14, respectively. The maximum velocity remains almost unchanged under a different number of baffles. While at the baffle, the fluid direction changes, and the streamline is cut off.

**Figure 18:**
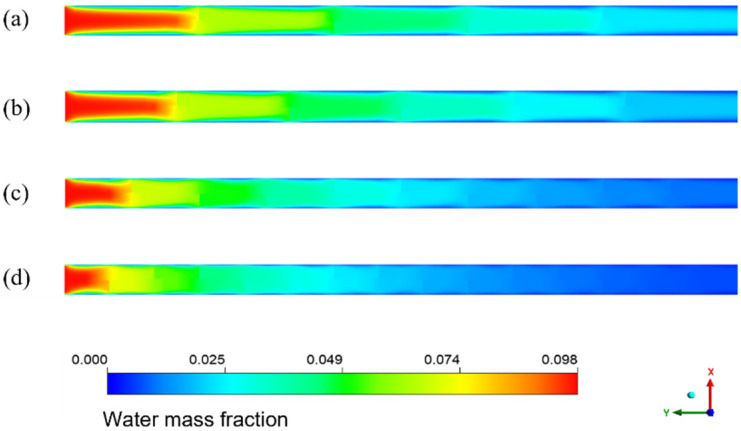
Water mass fraction distribution with different numbers of baffles in spiral flow membrane tube. (**a**) 4 baffles; (**b**) 5 baffles; (**c**) 9 baffles; (**d**) 14 baffles.

Figure 18 shows the distribution of the water mass fraction when the baffle number in the spiral flow membrane tube is 4, 5, 9, and 14, respectively. The water mass fraction decreases continuously along the flow direction. The contour changes near the baffle position because the water mass fraction decreases suddenly. The existence of baffles makes the flow direction of the sudden change, which enhances the disturbance to the fluid. It makes the separation process more sufficient and results in a fast decrease in the water mass fraction. When the baffle number increases, it can be found that the decreasing trend of water mass fraction is increasing, and the color of the separator outlet section is lighter. With a reduction in baffle spacing, the fluid disturbance is more intense, and the contact separation between fluids is sufficient. The color change trend in the membrane tube is more intense, and the outlet water mass fraction is lower.

**Figure 19:**
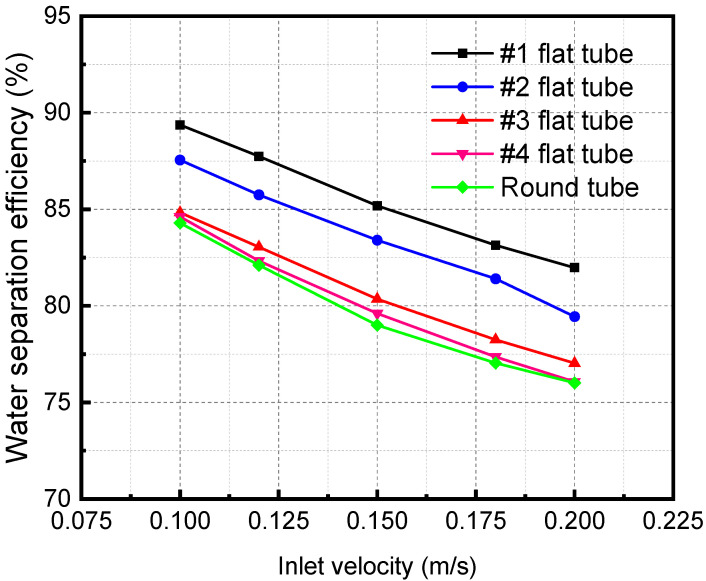
Effect of mixture inlet velocity on water mass fraction at the outlet.

The simulation results in Figure 19 show that when the number of baffles in the membrane tube is nine, the influence of different mixture inlet velocities on the outlet water mass fraction changes. As the mixture inlet velocity increases from 0.1 to 0.2 m/s, the outlet separation efficiency decreases correspondingly. At the same mixture inlet velocity, the separation efficiency of the #1 flat tube is the highest, while that of the circular tube membrane separator is the lowest. As the membrane tube flatness increases, the equivalent diameter of the membrane tube decreases gradually. The fluid flow in the tube becomes more intense, making the separation process between the mixture fluid and the absorption liquid more sufficient. Therefore, the water mass fraction at the outlet of the #1 flat tube is the lowest, and the separation effect is the best. Compared with the segmental baffles arranged in the membrane with an area of half of the flow cross-section area, the separation efficiency decreases, and the pressure drop in the membrane tube increases dramatically. The flow cross-section area in the membrane tube decreases, and the turbulence degree of the fluid increases. The energy consumption of the fluid flow in the tube increases, and the separation between the mixed fluid and the absorbent fluid becomes inadequate, reducing the membrane separation efficiency.

## 5. Conclusions

Coupled with the species transport model, the porous medium model, and the water absorption mathematical model, a three-dimensional shell-tube HFM separator model is established. The flow field in the membrane of the round tube and flat membrane separator with different flatness is simulated using the numerical simulation method. The flow field characteristics of several membrane tube separators at different inlet flow rates are compared, and the influence of different parameters on the membrane separator performance is analyzed. The flow field characteristics in the membrane tube and its influence on the performance of the membrane separator are discussed by adding a baffle plate and bow baffle to the tube side. The following conclusions are reached.

Under the same Reynolds number, an increase in the flat tube flatness will reduce the outlet water mass fraction, but the membrane tube pressure increases. Therefore, the membrane tube flatness cannot be increased without limit. Otherwise, the hydrodynamic consumption will be increased. With an increase in membrane tube flatness, the separation efficiency also increased. When the inlet velocity of the mixture is 0.1 m/s, the separation efficiency is 75.92%. The separation efficiency and the pressure drop inside the membrane separator increased when the membrane tube lengthened. The membrane tube length has the same effect as the flatness, but increasing the length will raise the production cost and difficulty of the membrane separator. Therefore, it is necessary to choose the appropriate length to construct the best structure of the membrane separator.

The mixed fluid meanders forward under the blocking effect of the bow baffle in the membrane tube and flows in a “Z” shape. Due to the existence of the baffle, the fluid flow direction changes dramatically, leading to increased energy consumption and significant pressure reduction in the membrane tube. The built-in torsional baffles can improve the mass transfer coefficient. The existence of torsional baffles makes the fluid flow generate vortex flow, which intensifies the turbulence, dramatically strengthens the mixing between the near surface area and the core area, reduces or even destroys the boundary layer, and promotes the separation of water in the mixed fluid. With the increased segmental baffle number in the membrane tube, the pressure drop in the tube and the separation efficiency increase continuously.

## Figures and Tables

**Table 1:** Structural parameters of membrane tube.

Name	Round Tube	#1 Flat Tube	#2 Flat Tube	#3 Flat Tube	#4 Flat Tube
Length, *l*/mm	180	180	180	180	180
Width, *w*/mm	1.60	2.05	1.88	1.76	1.67
Height, *h*/mm	1.60	1.20	1.30	1.40	1.50
Flatness, *e*/h	1.00	1.71	1.45	1.26	1.11
Hydraulic diameter, *d*/mm	1.00	0.88	0.94	0.98	1.00
Film thickness, *σ*/mm	0.3	0.3	0.3	0.3	0.3
Circulating sectional area, *S*/mm^2^	0.79	0.79	0.79	0.79	0.79

**Table 2:** Boundary conditions for numerical simulation of HFM [12].

Parameter Name	Numerical Value
Mixture inlet temperature, *T_m,i_*/°C	160
Mixture inlet pressure, *p_m,i_*/MPa	20
LiBr solution inlet temperature, *T_s,i_*/°C	160
LiBr solution inlet pressure, *p_s,i_*/MPa	20
LiBr solution inlet concentration, *X_s,i_*/wt%	65
SCO_2_ mass fraction in the mixture, *X_s,c_*/wt%	90.17
Water mass fraction in the mixture, *X_w_*/wt%	9.83
Water diffusion coefficient in sCO_2_, *D_w,sc_*/(m^2^/s)	7.69 × 10^−8^
Water diffusion coefficient in LiBr solution, *D_w,s_*/(m^2^/s)	3 × 10^−9^
LiBr solution dynamic viscosity, *μ_s_*/(Pa∙s)	5 × 10^−3^
LiBr solution density, *ρ_s_*/(kg/m^3^)	1638

## Data Availability

The authors confirm that the data supporting the findings of this study are available within the article.
